# The Enemy of My Enemy: Two Cases of De Novo Metastatic Small-Cell Neuroendocrine Prostate Cancer with BRCA2 Alterations and Brain Metastases

**DOI:** 10.3390/ijms27198453

**Published:** 2026-09-22

**Authors:** Mads Fallesen Kjer, Mads Hvid Poulsen, Sanne Kjær-Frifeldt, Marianne Ingerslev Holt, Mie Holm Vilstrup, Ahmed H. Zedan

**Affiliations:** 1 Department of Oncology, Vejle Hospital, University Hospital of Southern Denmark, 7100 Vejle, Denmark; 2 Department for Urology, Esbjerg and Grindsted Hospital, University Hospital of Southern Denmark, 6700 Esbjerg, Denmark; 3 Department of Regional Health Research, University of Southern Denmark, 5230 Odense, Denmark; 4 Department of Pathology, Vejle Hospital, University Hospital of Southern Denmark, 7100 Vejle, Denmark; 5 Department of Clinical Genetics, Vejle Hospital, University Hospital of Southern Denmark, 7100 Vejle, Denmark; 6 Department of Radiology and Nuclear Medicine, Esbjerg and Grindsted Hospital, University Hospital of Southern Denmark, 6700 Esbjerg, Denmark

**Keywords:** metastatic prostate cancer, neuroendocrine carcinoma, BRCA1/2 alterations, PARPis, brain metastases

## Abstract

De novo small-cell neuroendocrine prostate cancer (SCNE PC) is a rare and highly aggressive subtype of prostate cancer with limited treatment options. *BRCA2* alterations may increase sensitivity to platinum-based chemotherapy and PARP inhibition; however, evidence regarding their clinical relevance in SCNE PC is limited. We present two cases of de novo SCNE PC with distinct *BRCA2* alterations. Both patients presented with a similarly extensive disease burden and initially received carboplatin and etoposide (CE), but their clinical courses differed markedly. Patient 1 developed brain metastases three months after completing six cycles of CE and died shortly after rechallenge with CE. In contrast, Patient 2 responded to repeated CE rechallenges, followed by short-term disease control with topotecan and 18 months of disease control with the PARP inhibitor olaparib. He subsequently developed leptomeningeal carcinomatosis and died shortly thereafter, with an overall survival of 5 years and 8 months. These cases illustrate the highly aggressive clinical course of de novo SCNE PC. Despite the potential predictive value of *BRCA2* alterations, they may be associated with a higher incidence of brain metastases.

## 1. Background

Neuroendocrine prostate cancer (NEPC) is an uncommon variant of prostate cancer (PC) that typically presents with normal or slightly elevated prostate-specific antigen (PSA) levels and visceral metastases at diagnosis [1]. It occurs either as primary NEPC in approximately 2% of patients with de novo metastatic castration-sensitive PC (mCSPC) [2,3] or as treatment-emergent NEPC (t-NEPC) in approximately 25% of patients with metastatic castration-resistant prostate cancer (mCRPC) [4,5,6].

NEPC has an unfavorable prognosis, with a median overall survival (OS) rarely exceeding one year [7,8,9]. NEPC responds poorly to androgen deprivation therapy (ADT) and therefore progresses rapidly [8,10,11,12].

NEPC arises from prostate cancer cells through a process known as neuroendocrine transdifferentiation. Several studies have identified shared genomic rearrangements in prostatic adenocarcinoma and NEPC foci within the same patient [13,14,15]. Immunohistochemical (IHC) analyses of NEPC often demonstrate expression of neuroendocrine markers, including chromogranin A, synaptophysin, neuron-specific enolase, and/or CD56. However, the absence of all neuroendocrine markers has been documented in approximately 10% of patients with NEPC [16]. In addition, NEPC often shows negative IHC staining for the androgen receptor and PSA. The histological and molecular spectrum of NEPC includes pure small-cell neuroendocrine prostate cancer (SCNE PC), with a reported incidence of 2%, as well as mixed tumors containing prostatic adenocarcinoma and carcinoma with neuroendocrine features [2,17,18,19].

The breast cancer susceptibility genes *BRCA1* and *BRCA2* are involved in homologous recombination repair (HRR) of DNA double-strand breaks [20]. Loss of function of the tumor suppressor gene *BRCA2*, due to either mutation or homozygous deletion, impairs HRR and is associated with an increased risk of cancer [21], particularly breast, pancreatic, and prostate cancer [22]. The prevalence of pathogenic germline and somatic *BRCA2* alterations in patients with mCRPC is approximately 5% and 13%, respectively [23]. However, varying frequencies of *BRCA2* alterations (8% and 25%) have been reported in patients with NEPC [5,24].

The detection of *BRCA2* alterations in NEPC is important because it may have therapeutic implications for treatment with poly(ADP-ribose) polymerase inhibitors (PARPis) or platinum-based chemotherapy [23,25,26,27,28].

PARPis, either as monotherapy or in combination with androgen receptor pathway inhibitors (ARPIs), have been extensively investigated in patients with prostatic adenocarcinoma and *BRCA1/2* alterations, demonstrating a survival benefit in both mCSPC and mCRPC [29,30,31,32,33,34,35]. However, clinical trial evidence supporting their use in patients with NEPC is lacking. Various platinum-based regimens have been investigated in patients with NEPC and *BRCA2* [36,37].

The brain is an uncommon site of metastasis in PC, with a reported incidence of approximately 2% in prostatic adenocarcinoma [38] and up to 25% in NEPC [39]. Nevertheless, the incidence of brain metastases (BrMs) in patients with PC has increased in recent decades, which may be partly attributable to improved survival.

Here, we describe the management of two patients at our center who were diagnosed with de novo metastatic NEPC harboring *BRCA2* alterations.

## 2. Case Presentation

### 2.1. Case 1

A 62-year-old male presented with symptoms of fatigue, unintentional weight loss over several months, and dyspnea. He had no significant medical history and no known family history of cancer.

Given the initial suspicion of primary lung cancer, an [^18^F]fluorodeoxyglucose positron emission tomography/computed tomography (FDG PET/CT) scan was performed. The scan revealed a right pulmonary mass, FDG-avid right hilar lymphadenopathy, suspected lesions in the pancreatic head and liver, and multiple skeletal metastases. The prostate gland was enlarged and showed focal FDG uptake (Figure 1).

An endobronchial ultrasound-guided biopsy of the right hilar lymph nodes was performed. Cytological examination revealed numerous malignant tumor cells arranged in small cohesive clusters, with nuclear crowding, molding, and overlap, hyperchromatic nuclei, and predominantly scant cytoplasm. IHC staining was positive for CK AE1/AE3, CK7, TTF-1, INSM1, synaptophysin, chromogranin A, and CD56, and negative for NKX3.1, PSA, CK20, and CDX2. The Ki-67 proliferation index was approximately 80%. Overall, the morphological and IHC findings were consistent with high-grade small-cell neuroendocrine carcinoma. Despite strong TTF-1 expression, the primary site could not be determined with certainty.

At presentation, the patient had lower urinary tract symptoms (LUTSs), including urinary retention requiring catheterization. Digital rectal examination (DRE) revealed a firm, irregular prostate, raising suspicion of de novo mCSPC. The baseline PSA level was 6.3 µg/L.

Treatment with carboplatin and etoposide (CE) was initiated before confirmation of the primary site because of the aggressive disease and extensive metastatic involvement. Carboplatin was administered at an AUC of 4, and etoposide at 75% of the standard dose (240 mg/m^2^). The following day, transperineal prostate biopsies were performed, and ADT with degarelix (Firmagon, 240 mg) was initiated.

Histological examination of the prostate biopsies revealed a mixed tumor, with conventional acinar adenocarcinoma in two biopsies and small-cell neuroendocrine carcinoma in eight biopsies. The latter showed extensive areas of tumor cells arranged in large confluent nests, predominantly with hyperchromatic nuclei and scant cytoplasm. Immunohistochemically, these cells were negative for PSA and positive for synaptophysin and CD56. The Ki-67 proliferation index was 85–90% (Figure 2).

The patient was staged as having cT4N1M1c disease and received six cycles of CE, with radiological assessment by NaF-PET/CT after every two cycles. After the first two cycles, imaging showed a partial response, with regression of the pulmonary, hepatic, and osseous metastatic lesions. PSA levels decreased to 0.60 µg/L after two cycles of CE and to 0.32 µg/L after six cycles.

TruSight Oncology 500 (TSO500) analysis was successfully performed and identified a pathogenic *BRCA2* variant (p.Y1710*) with a variant allele frequency (VAF) of 89%. In addition, a pathogenic *BRAF* variant (*p.K601E*) and a likely pathogenic *TP53* variant (p.V143Hfs*26) were detected. Copy-number analysis indicated loss of one *BRCA2* copy. The tumor was microsatellite-stable (MSS) and had a low tumor mutational burden (TMB) of 4.7 mutations/Mb.

After completing six cycles of CE, the patient was advised to take a treatment break while continuing ADT, with repeat imaging performed three months later. Three months into the treatment break, he presented to the emergency department with acute confusion and left upper limb weakness. Brain MRI revealed multiple supratentorial and infratentorial BrMs (Figure 3). Clinically, he showed symptoms of progression, with worsening skeletal-related pain, although imaging showed otherwise stable extracranial disease.

The patient received whole-brain radiotherapy at a dose of 30 Gy in 10 fractions. The indication for further systemic treatment and the available options—reintroduction of CE or initiation of the PARP inhibitor olaparib—were discussed with the patient and his family. The patient preferred rechallenge with CE.

Eight days after the first cycle of CE rechallenge, the patient presented to the emergency department with significant dyspnea and back pain. On admission, he was in septic shock, with hypotension, tachypnea, and severe lactic acidosis (lactate > 15 mmol/L). Despite intensive supportive treatment, including oxygen therapy, intravenous fluids, and bicarbonate administration, his condition deteriorated rapidly, and he died two hours after admission.

His overall survival from diagnosis was 8 months and 3 days.

The patient was referred for genetic counseling during treatment but died before a blood sample could be collected for genetic analysis.

A summary of the patient’s treatment is illustrated in Figure 4.

### 2.2. Case 2

A 66-year-old male presented to the emergency department with symptoms of nausea, vomiting, and abdominal pain. He reported unintentional weight loss of 3.5 kg over several weeks. The patient had no notable medical history and no family history of cancer.

The patient had no LUTSs. On DRE, the prostate was firm and irregular. CT of the thorax, abdomen, and pelvis revealed multiple liver and bone metastases (Figure 5).

At diagnosis, the PSA level was 0.45 µg/L, and the patient was staged as having cT3N1M1c disease. Transrectal ultrasound-guided prostate biopsies and liver biopsies were performed to establish the primary site.

All six prostate biopsies from both lobes showed pure small-cell neuroendocrine carcinoma, with no conventional adenocarcinoma component. The tumor diffusely infiltrated the tissue in irregular nests and cords. The tumor cells exhibited a high nuclear-to-cytoplasmic ratio, scant cytoplasm, closely packed hyperchromatic nuclei with nuclear molding, and scattered mitotic figures. Immunohistochemically, the tumor cells were positive for synaptophysin, chromogranin A, and CD56, with a Ki-67 proliferation index of approximately 100%.

A subsequent biopsy from a liver metastasis showed similar morphology, with confluent nests of tumor cells displaying a high nuclear-to-cytoplasmic ratio, hyperchromatic nuclei, scant cytoplasm, nuclear debris, and nuclear molding. The tumor cells were negative for PSA and positive for synaptophysin, with focal positivity for chromogranin A and CD56. The Ki-67 proliferation index was again close to 100% (Figure 6), supporting the diagnosis of metastatic small-cell neuroendocrine carcinoma.

Chemotherapy was initiated promptly with a planned CE regimen. The first cycle consisted of carboplatin monotherapy at an AUC of 4 because the patient’s bilirubin level exceeded 100 µmol/L. Etoposide was introduced at the full dose from the second cycle.

ADT was initiated on the day of diagnosis with degarelix (Firmagon, 240 mg). After the initial dose, treatment was switched to leuprorelin (Eligard, 45 mg).

After three cycles, radiological assessment by NaF-PET/CT showed a partial response, with a marked reduction in liver metastases and increased sclerosis of the bone metastases (Figure 7).

From November 2020 to September 2023, the patient received three rechallenges with six-cycle courses of CE, each consisting of six cycles of CE, alternating with six-month treatment breaks. During the third rechallenge, disease progression was observed after the sixth cycle, and treatment was switched to second-line chemotherapy with topotecan. After six cycles of topotecan, imaging showed stable disease.

A follow-up scan three months after the completion of topotecan showed the progression of metastases in the liver, adrenal glands, and bones. Comprehensive genomic profiling of the initial prostate biopsies from 2020 using the TSO500 panel detected no pathogenic variants in *BRCA1* or *BRCA2*. Copy-number analysis estimated zero copies of both *BRCA2* and *AR*, suggesting a possible loss of both genes. Additional copy-number alterations were identified in *MYCL, MDM4, MYC, FGF19, FGF9*, and *LAMP1*. The tumor was microsatellite-stable (MSS) and had a low tumor mutational burden (TMB) of 1.6 mutations/Mb. Concurrently, the patient underwent genetic counseling and exome-based germline analysis of 77 genes, including *BRCA1*, *BRCA2*, and genes associated with Lynch syndrome, with a normal result.

Following identification of the *BRCA2* deletion, the patient received olaparib. Treatment resulted in a partial response and maintained disease control for 18 months before progression.

The patient subsequently underwent repeat comprehensive genomic profiling of a fresh biopsy from a liver metastasis. Paired tumor-normal whole-genome sequencing and RNA sequencing were performed, confirming the previously documented somatic loss of one *BRCA2* allele and, in addition, a somatic inversion of *EEF1DP3*-*BRCA2* affecting the transcript of *BRCA2*, leading to presumed loss of function of the other allele and therefore classification as likely pathogenic. Hence, it was concluded that the tumor exhibited biallelic loss of *BRCA2*.

No national experimental protocols were available for patients with NEPC harboring somatic *BRCA2* loss whose disease had progressed during PARPi treatment. Given the patient’s preference and his favorable responses to previous CE rechallenges without substantial adverse effects, a fourth rechallenge with the same regimen was initiated. After six cycles of CE, imaging showed stable disease, followed by a planned three-month treatment break.

Two months after completing the fifth course of CE, the patient experienced a generalized tonic–clonic seizure. Brain MRI showed diffuse supratentorial and infratentorial leptomeningeal contrast enhancement, suggestive of leptomeningeal carcinomatosis (Figure 8).

Cerebrospinal fluid analysis was negative for infection and paraneoplastic antibodies but revealed malignant cells, confirming leptomeningeal dissemination. Given the patient’s rapid neurological decline and worsening performance status, further oncological treatment was considered inappropriate, and best supportive care was provided.

He died three weeks later. His overall survival from diagnosis was 5 years and 8 months.

A summary of the patient’s treatment is illustrated in Figure 9.

## 3. Discussion

In this report, we describe the oncological management of two patients with de novo SCNE PC and distinct *BRCA2* alterations. Patient 1 had a pathogenic *BRCA2* variant and loss of one *BRCA2* copy. Patient 2 initially showed a biallelic loss of *BRCA2*. Patient 2 had an exceptionally prolonged clinical course, with an overall survival of 5 years and 8 months, repeated sensitivity to CE, and 18 months of disease control with olaparib. Despite the marked difference in overall survival, both patients died shortly after being diagnosed with BrMs: parenchymal brain metastases in Patient 1 and leptomeningeal carcinomatosis in Patient 2.

NEPC is an uncommon and fatal variant of PC [11]. Although de novo NEPC is relatively rare, the incidence of t-NEPC in CRPC has increased in recent decades, likely because of the widespread use of ARPIs [11,40].

Germline and/or somatic *BRCA1/2* alterations are associated with aggressive PC, an increased likelihood of disease progression, and shorter survival [41]. The predominant genomic alterations in NEPC are *ATM/BRCA* variants, occurring in 17% of cases, with *BRCA2* alterations accounting for 11% [42]. This prevalence is nearly threefold higher than that observed in patients with metastatic prostatic adenocarcinoma [43]. Approximately 5% of *BRCA1/2* gene alterations in mCRPC are reported to be of germline origin, whereas 11% are reported to be somatic [44].

Due to the limited availability of prospective clinical trial data, researchers have yet to establish standard management strategies for NEPC. Platinum-based chemotherapy is the primary treatment option for patients presenting with NEPC, typically producing an initial response, with a median progression-free survival of 2–8 months and a median OS of approximately 12 months [2,45,46,47].

However, effective treatment options after first-line chemotherapy are limited in NEPC, as in small-cell lung cancer (SCLC) [38].

Alterations in DNA damage repair genes, such as *BRCA2*, increase tumor sensitivity to PARPis and platinum-based chemotherapy. Platinum compounds induce DNA cross-linking, resulting in the death of *BRCA1/2*-deficient tumor cells with impaired DNA repair. Olaparib, a selective PARPi that inhibits both PARP1 and PARP2, induces tumor cell death through synthetic lethality by exploiting HRR deficiency [48,49]. Platinum-based chemotherapy has not been prospectively evaluated in patients with PC and HRR-related gene alterations; therefore, consensus on its efficacy relative to standard therapies has not yet been reached [50,51,52]. In cases of ovarian and pancreatic cancers associated with *BRCA1/2* alterations, the standard of care includes platinum induction therapy followed by maintenance with PARPis [53,54]. Conversely, in *BRCA1/2*-mutated metastatic breast cancer, platinum chemotherapy is recommended as a first-line treatment, whereas PARPis are designated for relapsed cases [55].

The optimal sequencing of platinum-based chemotherapy and PARPis, as well as the extent of cross-resistance between these treatments, has been investigated in patients with *BRCA1/2*-altered PC; however, the available evidence remains conflicting [56,57,58,59]. Slootbeek et al. reported four cases of mCRPC with *BRCA2* alterations that responded favorably to first-line platinum-based chemotherapy and subsequent PARPi treatment [59]. Mota et al. evaluated platinum-based chemotherapy after PARPi treatment in six patients with mCRPC and DNA damage repair gene alterations and observed a sustained response [56]. Miyazawa et al. reported two cases of NEPC with *BRCA2* alterations that responded well to second-line olaparib [57]. Pandya et al. reported a survival of 18 months in a patient with NEPC harboring a germline *BRCA2* alteration who received olaparib maintenance therapy [60]. However, concerns regarding cross-resistance have arisen in patients with PC who progress on platinum-based chemotherapy following PARPi treatment [58], similar to observations in patients with ovarian cancer [61].

The clinical role of ADT in patients with NEPC remains controversial and is influenced by the histopathological composition of the tumor. In patients with pure NEPC, the therapeutic benefit of ADT is uncertain, given the typically limited dependence of neuroendocrine tumor cells on androgen receptor signaling. In contrast, the presence of a concurrent conventional acinar adenocarcinoma component may provide a rationale for continuing ADT as part of the treatment strategy.

In Case 1, the identification of a conventional acinar adenocarcinoma component supported the continuation of ADT. In Case 2, however, no conventional adenocarcinoma component was identified in the available prostate biopsy specimens. In retrospect, therefore, the rationale for incorporating ADT into the systemic treatment regimen alongside chemotherapy was limited in this case.

The incidence of BrMs is higher in less common histological subtypes of PC than in prostatic adenocarcinoma, with reported rates of 10% in SCNE PC and 25% in NEPC [39]. *BRCA* genes alterations have been associated with a higher risk of BrMs both in patients with breast cancer and patients with ovarian cancer [62,63]. Whether *BRCA2* alterations similarly increase the risk of central nervous system involvement in NEPC remains unexplored; these two cases are hypothesis-generating and cannot establish such an association.

The median interval from PC diagnosis to the development of BrMs is approximately 42 months, with a range of 6–90 months [64]. The intervals observed in our two cases were within this range: the first patient developed cerebral metastases approximately six months after the initial PC diagnosis, whereas leptomeningeal carcinomatosis was confirmed radiologically and cytologically in the second patient approximately 60 months after diagnosis.

The distinct molecular characteristics identified in the two patients presented in this study may, at least in part, account for the observed differences in treatment response. In particular, the biallelic loss of *BRCA2* identified in Patient 2 may have contributed to the pronounced treatment sensitivity. However, given the limited evidence from a single case, a definitive causal relationship between *BRCA2* loss and the observed therapeutic response cannot be established.

The reported median OS after diagnosis of BrMs in patients with metastatic PC is approximately nine months, irrespective of treatment [65]. Survival after BrM diagnosis was substantially shorter in both patients described here.

In Case 1, brain metastases developed in the absence of extracranial progression. This pattern has been reported previously [66,67].

The optimal systemic treatment for patients with PC and BrMs has not been established, as these patients are usually excluded from clinical trials. Taxanes differ in their ability to cross the blood–brain barrier (BBB). Docetaxel penetration across the BBB is limited by drug efflux pumps [68], whereas cabazitaxel shows greater permeability in rodent models, although the clinical relevance of its intracranial antitumor activity in PC remains uncertain [69]. ARPIs achieve varying intracranial concentrations. Apalutamide and enzalutamide reach concentrations associated with a small risk of seizures [70,71,72]. Abiraterone can cross the BBB, but evidence of clinically meaningful intracranial antitumor activity is lacking [73]. With a few exceptions, such as niraparib, PARPis, including olaparib, rucaparib, and talazoparib, generally have limited ability to cross the BBB [74,75,76,77,78,79].

## 4. Conclusions

NEPC is an uncommon variant of PC that is generally associated with an unfavorable prognosis. The present cases demonstrate markedly divergent clinical courses in patients harboring distinct *BRCA2* alterations. In Patient 2, the repeated sensitivity to platinum-based chemotherapy, prolonged disease control with olaparib, and comparatively extended overall survival may suggest a potential association between biallelic *BRCA2* loss and enhanced treatment sensitivity. However, given the single-case nature of this observation, a causal relationship cannot be established. The development of BrM in both patients may indicate a clinically relevant pattern of disease progression among patients with NEPC and *BRCA2* alterations. Nevertheless, any potential association between *BRCA2* alterations and an increased propensity for BrM remains hypothesis-generating and warrants further investigation in larger patient cohorts.

## Figures and Tables

**Figure 1:**
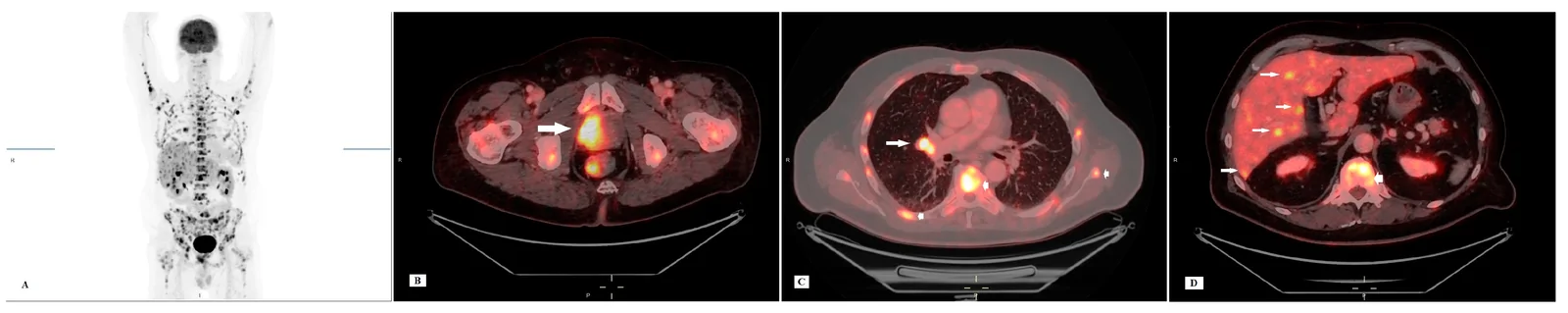
[^18^F]FDG PET/CT findings. (**A**) Multiple FDG-avid metastatic lesions. (**B**) Intense focal uptake in the prostate (arrow). (**C**) Intense focal uptake in the tumor/lymph nodes at the right pulmonary hilum (arrow) and a bone metastasis (short arrow). (**D**) Multiple foci of uptake in the liver (arrows) and a bone metastasis (short arrow).

**Figure 2:**
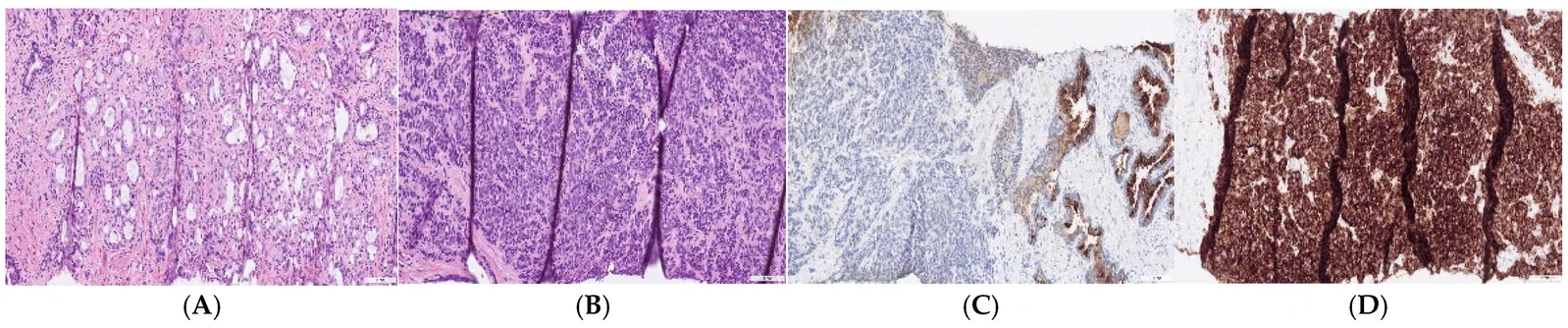
Histological and immunohistochemical features of the prostate tumor. (**A**) Conventional prostatic adenocarcinoma. (**B**) Tumor cells arranged in large confluent nests, predominantly displaying hyperchromatic nuclei and scant cytoplasm. (**C**) Tumor cells showing negative PSA staining, with adjacent non-neoplastic prostatic glands serving as a positive internal control. (**D**) Positive immunoreactivity for synaptophysin. Original magnification ×20.

**Figure 3:**
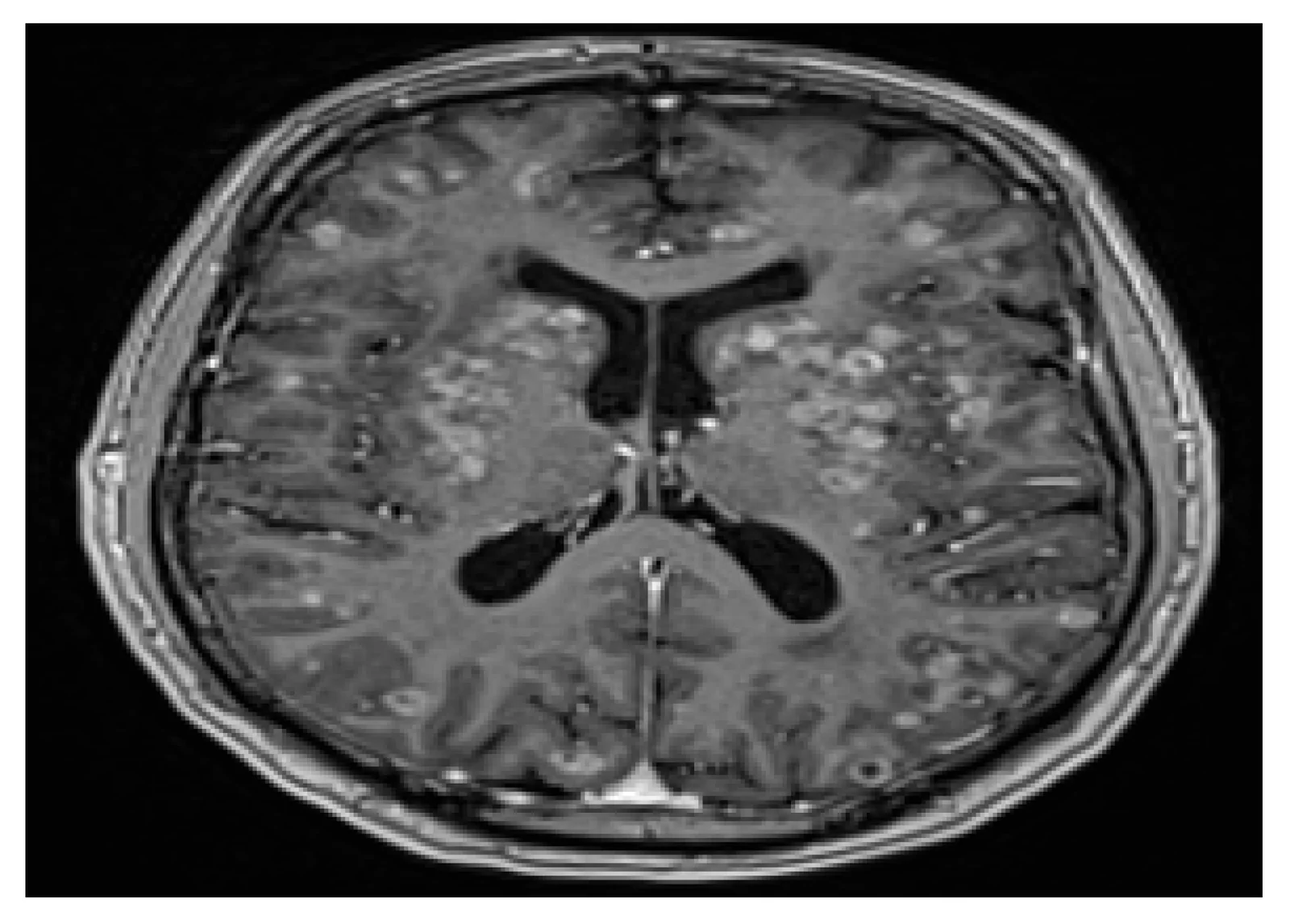
Brain MRI showing multiple small ring-enhancing lesions involving the basal ganglia and bilateral cortical–subcortical junctions, consistent with metastases, on an axial contrast-enhanced 3D T1-weighted image.

**Figure 4:**
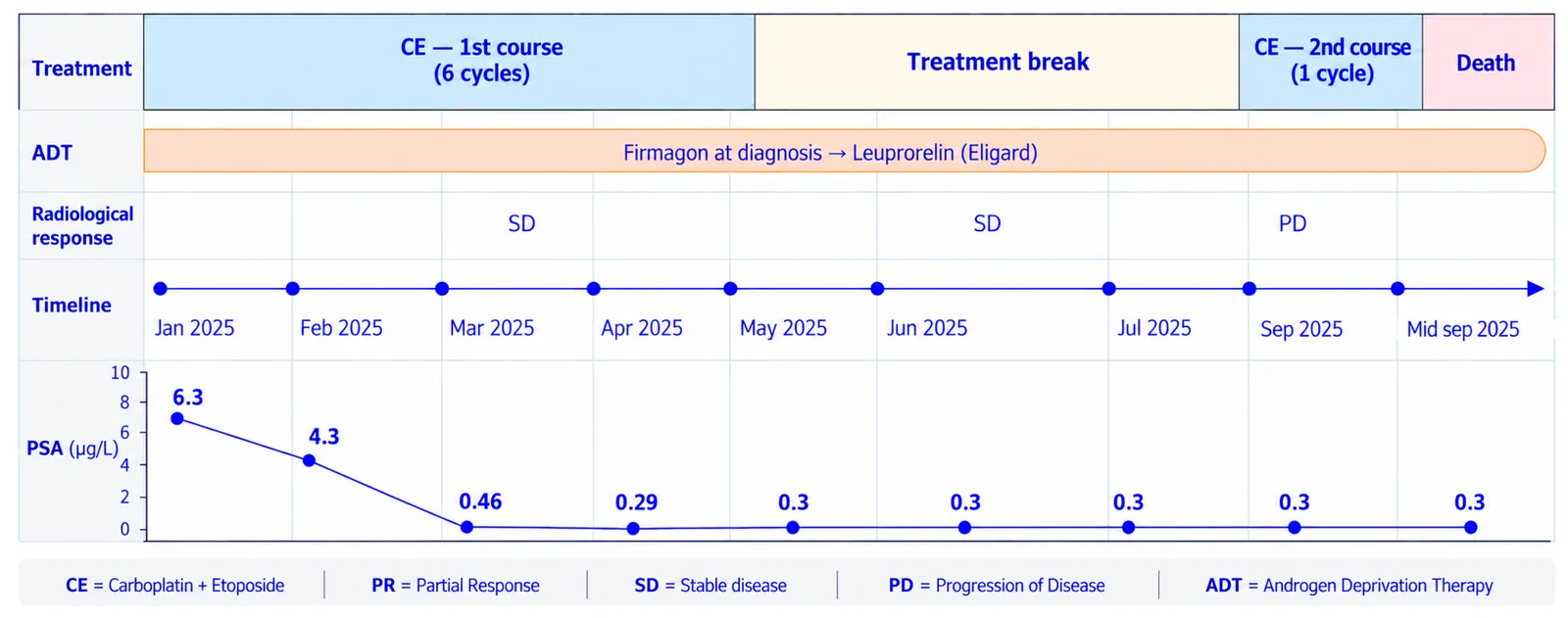
Timeline for the treatment course for case 1, including both biochemical and radiological responses.

**Figure 5:**
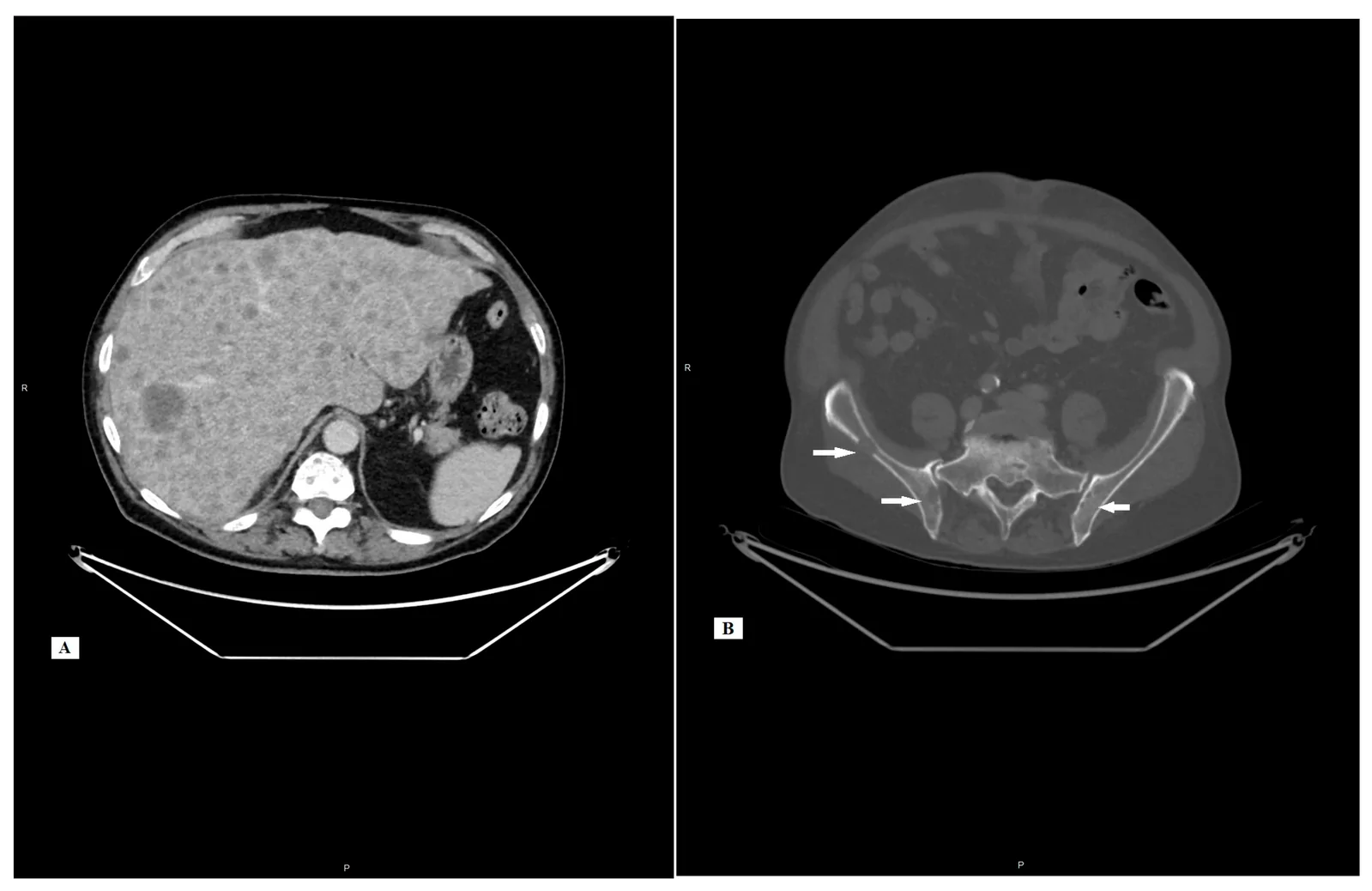
Baseline CT showing multiple liver metastases (**A**) and osteolytic bone metastases ((**B**), arrows).

**Figure 6:**
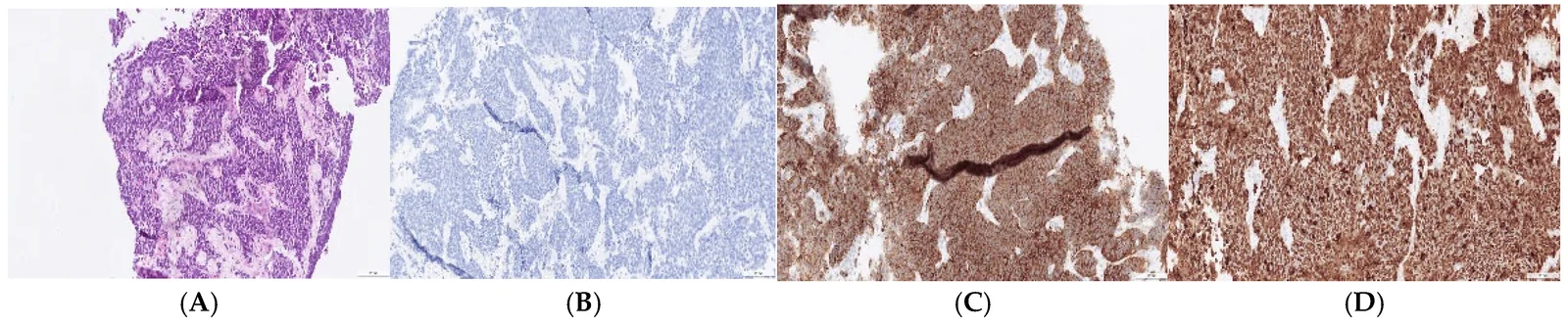
Histological and immunohistochemical features of the liver metastasis. (**A**) Tumor tissue composed of confluent nests of cells with a high nuclear-to-cytoplasmic ratio, hyperchromatic nuclei, scant cytoplasm, nuclear debris, and nuclear molding. (**B**) Tumor cells showing negative PSA staining. (**C**) Strong positive immunoreactivity for synaptophysin. (**D**) Ki-67 immunohistochemistry demonstrates a proliferation index approaching 100%. Original magnification ×20.

**Figure 7:**
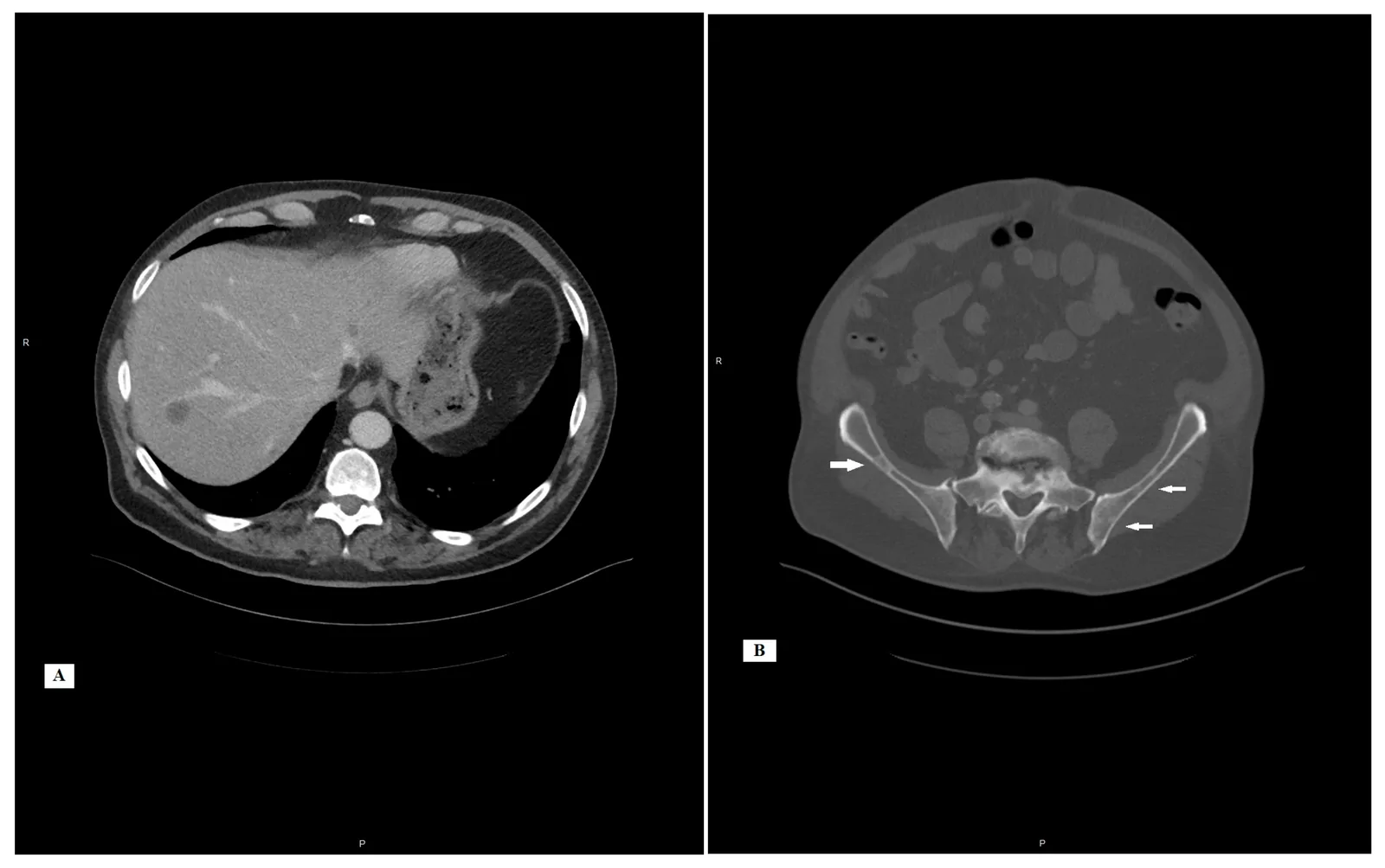
CT scan after three cycles of chemotherapy showing a partial response, with a marked reduction in liver metastases (**A**) and sclerotic changes in bone metastases consistent with healing ((**B**), arrows).

**Figure 8:**
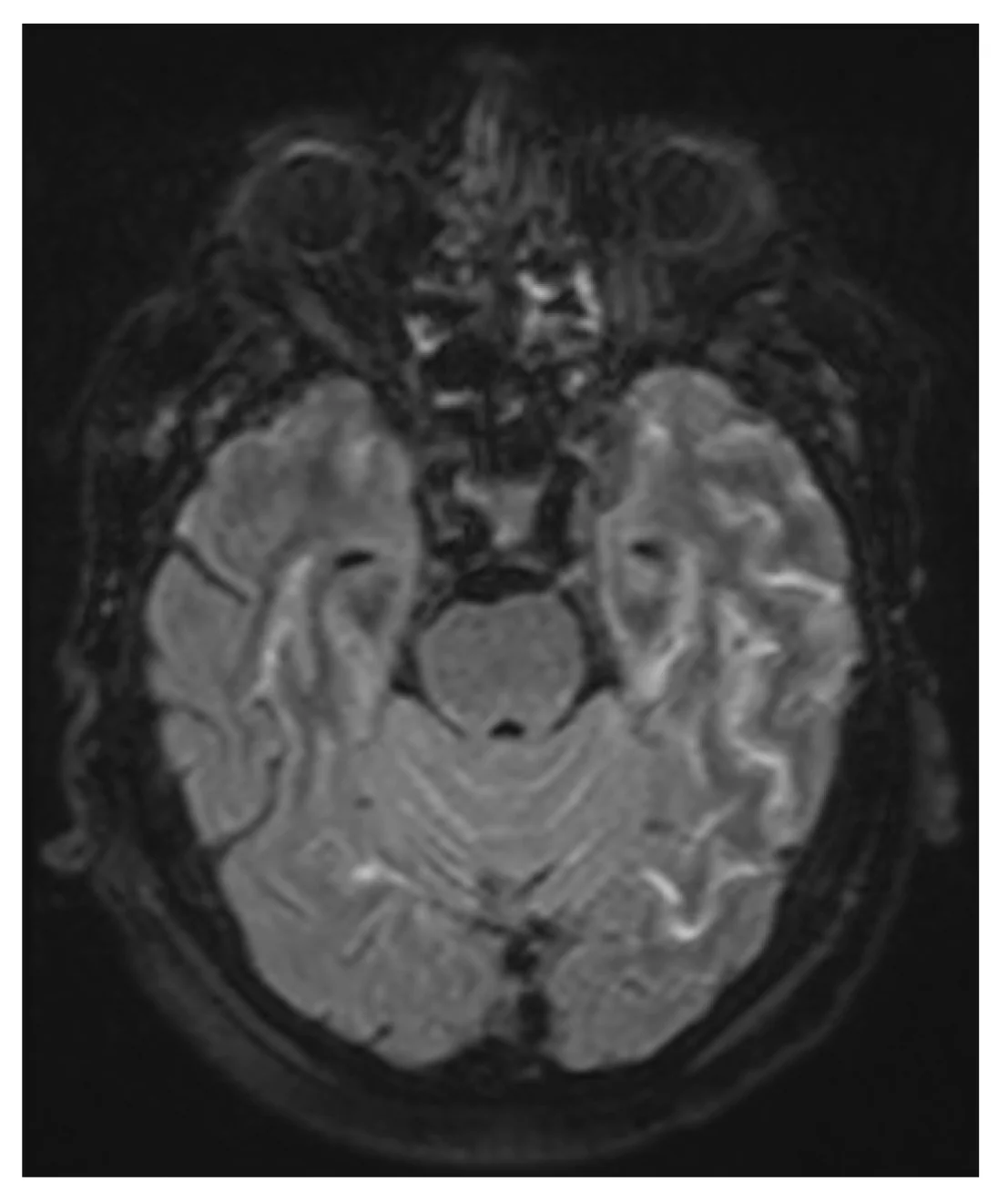
Brain MRI showing diffuse supratentorial and infratentorial leptomeningeal contrast enhancement on an axial contrast-enhanced 3D fluid-attenuated inversion recovery (FLAIR) image, consistent with leptomeningeal carcinomatosis.

**Figure 9:**
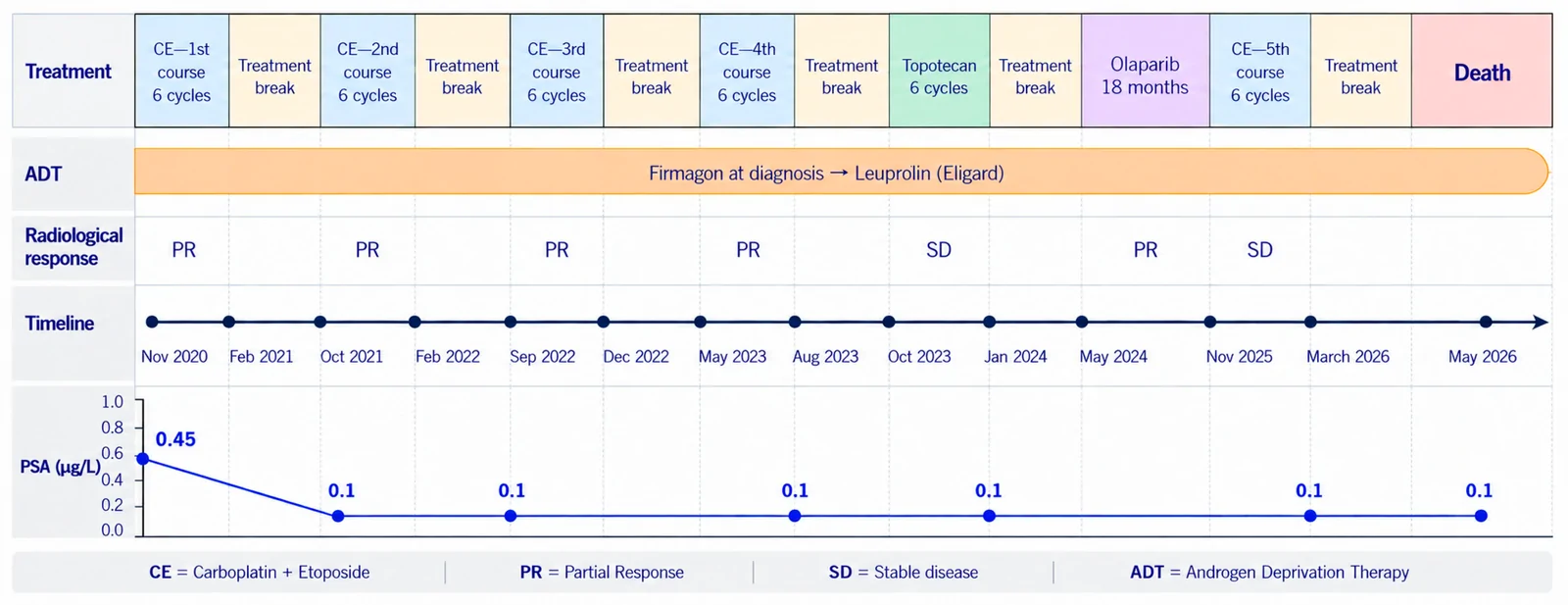
Timeline for treatment course for case 2, including both biochemical and radiological response.

## Data Availability

The original contributions presented in this study are included in the article. Further inquiries can be directed to the corresponding author.
